# *Bacillus coagulans* GBI-30, 6086 limits the recurrence of *Clostridium difficile*-Induced colitis following vancomycin withdrawal in mice

**DOI:** 10.1186/1757-4749-4-13

**Published:** 2012-10-22

**Authors:** Leo R Fitzpatrick, Jeffrey S Small, Wallace H Greene, Kelly D Karpa, Sean Farmer, David Keller

**Affiliations:** 1 Department of Pharmacology, Penn State College of Medicine, 1214 Research Boulevard, Hummelstown, PA, 17036, USA; 2 Department of Pathology, Penn State College of Medicine, PO Box 850, Hershey, PA, 17033, USA; 3 Ganeden Biotech Inc., 5800 Landerbrook Drive, Suite 300, Mayfield Heights, OH, 44124, USA

**Keywords:** *Clostridium difficile*, GanedenBC30, Probiotics, Colitis, Mice

## Abstract

**Background:**

Recently, we found that the probiotic strain *Bacillus coagulans* GBI-30, 6086 (GanedenBC^30^) improved indices of *Clostridium difficile (C. difficile)*-induced colitis in mice (Fitzpatrick et al., *Gut Pathogens*, 2011). Our goal was to determine if BC30 could also prevent the recurrence of *C. difficile*-induced colitis in mice, following initial treatment with vancomycin. During study days 0 through 5, mice were treated with antibiotics. On day 6, the *C. difficile* strain VPI 10463 was given by oro-gastric gavage at ≈ 5x10^4^ CFU to induce colitis. Mice were treated on study days 6 to 10 with vancomycin (50 mg/kg) (vanco) or vehicle (saline) by gavage. On days 10 to16, mice were dosed by gavage with saline vehicle or BC30 (2 x 10^9^ CFU per day). Mice were monitored for mortality, weight loss and diarrhea. On study days 14, 16 and 17, stools and colons were collected for analyzing other parameters of colitis.

**Results:**

The mean stool consistency score in Vehicle/C.difficile/Vanco mice increased from 0.4 (day 10) to a range of 1.1 to 1.4 (days 14 to 17), indicating the recurrence of colitis. On days 13 through 17, the stool consistency scores for the vancomycin/BC30 mice were significantly lower (p< 0.05) than for the vancomycin/vehicle cohort of animals. On day 17, 88.9% of mice treated with BC30 had normal stools, while this value was 0% with vehicle treatment (p value = 0.0004). Colonic myeloperoxidase (Units/2 cm colon) was significantly (p < 0.05) reduced from 4.3 ± 0.7 (Vehicle/C.difficile/Vanco) to 2.6 ± 0.2 (BC30/C. Difficle/Vanco). The colonic histology score and Keratinocyte derived-chemokine level in the colon were also lower in BC30 treated mice.

**Summary:**

In BC30-treated mice, there was evidence of better stool consistency, as well as improved biochemical and histological indices of colitis, following initial treatment of animals with vancomycin.

**Conclusion:**

BC30 limited the recurrence of *CD*-induced colitis following vancomycin withdrawal in mice.

## Background

*Clostridium difficile* (*C. difficile*) infection (CDI) is a very common cause of health-care associated diarrhea and colitis [1]. Moreover, CDI is associated with significant morbidity, as well as increased health care costs [2]. The spectrum of *C. difficile* associated disease (CDAD) ranges from mild antibiotic associated diarrhea to severe and life threatening pseudomembranous colitis [3]. CDAD is caused by the actions of two toxins (toxin A and toxin B), which are produced by pathogenic strains of *C. difficile*[4,5]. Toxin A results in the activation of three transcription factors (NF- kB, AP1 and CREB). NF-kB (nuclear factor-kappa B) is involved in chemokine production, and also plays a role in colonocyte apoptosis [6,7]. AP-1 (activator protein-1) plays a role in IL-8 production in response to stimulation of colonocytes with toxin A [8]. CREB (Cyclic-AMP Response Binding Protein) is critical for the production of prostaglandin E_2_ via inducible cyclooxygenase-2 (COX-2) [9]. This prostaglandin plays an important role in the fluid secretion and diarrhea associated with CDAD.

CDAD is often treated successfully with standard antibiotics such as vancomycin (vanco) or metronidazole [10,11]. However, recurrence occurs in at least 20% of patients [11]. Some clinical studies have focused on combined treatment with vancomycin and probiotics such as *Saccharomyces boulardii* for the treatment of recurrence [12-15]. Therefore, the use of probiotics, for prevention of recurrent disease, may be attractive as part of the overall therapeutic strategy for CDAD [12-15].

*Bacillus coagulans* GBI-30, 6086 (GanedenBC^30^) is a spore-forming probiotic strain that is resistant to extreme temperatures and survives in the gut environment [16]. BC30 was shown to have anti-inflammatory and immunomodulatory effects *in vitro* and *in vivo*[17,18]. Previously, we reported that BC30 improved various parameters of C. difficile-induced colitis in mice [18]. Additionally, BC30 prolonged the survival time in C. difficile-infected mice [18]. While the initial research focused on primary treatment of C. diifficile, this study reached the ability to prevent re-occurrences of C. *Difficile* infection following withdrawal of Vancomycin.

Recently, other investigators have described the recurrence of CDAD following vancomycin withdrawal in mice [10,19]. Overall, recurrence is associated with some evidence of disease (weight loss, diarrhea), as well as typical histological evidence of CDAD [10,19]. With knowledge of this previous scientific information, the goal of our study was to determine if BC30 could prevent recurrence of *CD*-induced colitis following vancomycin withdrawal in mice.

## Results

### Effects of BC30 on mouse survival and body weight, as well as the presence of *C. difficile* infection and toxins

**Figure 1:**
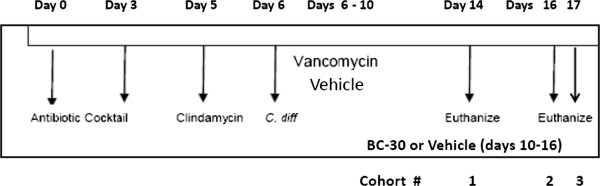
**Study overview.** The key events associated with the *Clostridium difficile* induced colitis mouse model are shown. On study days 0 through 3, C57BL/6 mice received an antibiotic mixture of kanamycin, gentamicin, colistin, metronidazole and Vanco in the drinking water, followed by clindamycin (10 mg/kg, i.p., on day 5). On day 6, the C*. difficile* strain VPI 10463 was given by oro-gastric gavage at ≈ 5x10^4^ CFU to induce colitis. Mice were treated on study days 6 to 10 with Vanco (50 mg/kg) or vehicle (saline) by gavage. On days 10 to 16, mice were dosed by gavage with vehicle (50% maltodextrin/saline, n=29) or BC30 (2 x 10^9^ CFU per day, n=28). One negative control group of mice (n=6) was dosed with vehicle, but did not receive *C. difficile*, while a positive control group (initial n of 8) received *C. difficile* but not Vanco. Mice were monitored daily (days 6 to 17) for mortality, weight loss and stool consistency. On study days 14, 16 and 17, stools and colons were collected for further analyses.

Figure 1 shows an overview of the key events associated with the *C. difficile* recurrence model that we used for this study. Cumulative survival rates in the study were: 100% (Vehicle/No *C. difficile*), 87.5% (Vehicle/*C. difficile*/No Vanco), 100% (Vehicle/*C. difficile*/Vanco) and 100% (BC30/*C. difficile*/Vanco). No statistically significant differences were found for mouse survival.

**Figure 2:**
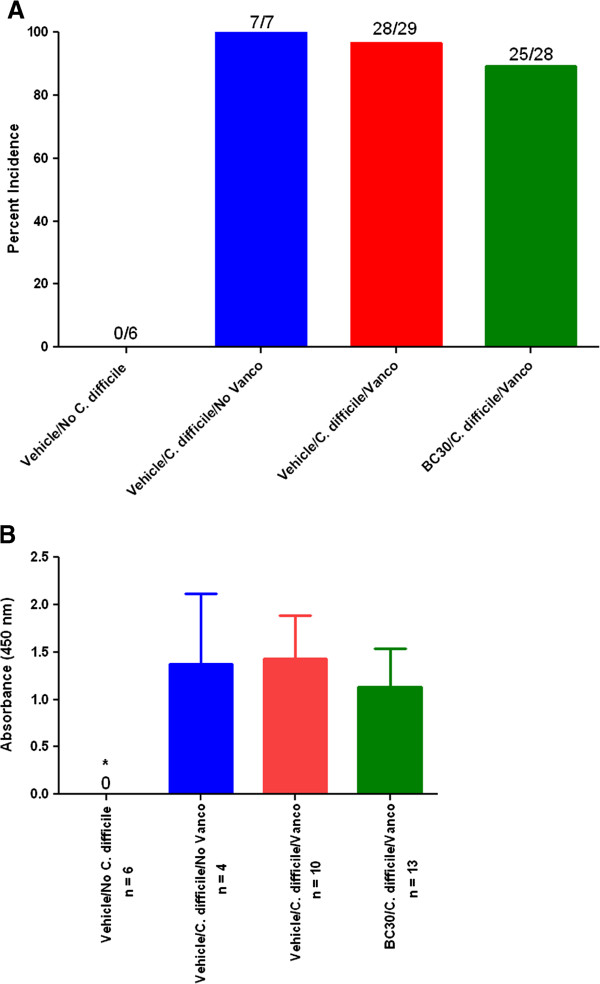
**Infection and toxin data. A**) The percentages of animals positive for *C. difficile* in the stool were determined by ELISA on study day 14, 16 and 17. * indicates p< 0.05 vs. all other *C. difficile* infection groups. **B**) Toxin A/B levels were determined in a semi-quantitative fashion with an appropriate ELISA kit, as described in the *Methods* section. The values in the graph represent absorbance readings at 450 nm. * indicates p< 0.05 vs. all other *C. difficile* infection groups.

The incidence rates of C. difficile infection from study days 14, 16 and 17 were: 0% (0/6, Vehicle/No *C. difficile*), 100% (7/7, Vehicle/*C. difficile*/No Vanco), 97% (28/29, Vehicle/*C. difficile*/Vanco) and 89% (25/28, BC30/*C. difficile*/Vanco) [Figure 2A]. The percentages of toxin A/B positive stools from these study days were: 0% (Vehicle/No *C. difficile*), 57% (Vehicle/*C. difficile*/No Vanco), 41% (Vehicle/*C. difficile*/Vanco) and 64% (BC30/*C. difficile*/Vanco). However, the semi-quantitative determination of toxin A/B levels (n = 6–13 per treatment group) showed increased absorbance readings (1.429 ± 0.456) from the stools of *Vehicle/C. difficile*/Vanco treated mice, as compared to absorbance readings (1.128 ± 0.410) from stools of BC30/*C. difficile*/Vanco treated animals [Figure 2B].

The mean body weights (grams) of mice on study day 6 were: 20.7 ± 0.5 (Vehicle/No *C. difficile*), 21.7 ± 0.6 (Vehicle/*C. difficile*/No Vanco), 21.8 ± 0.3 (Vehicle/*C. difficile*/Vanco) and 21.9 ± 0.3 (BC30/*C. difficile*/Vanco). Of note, surviving Vehicle/*C. difficile*/No Vanco treated mice did transiently lose an average of 1.1 grams between study days 7 and 9. On study day 17, the mean body weights (grams) of remaining mice (n = 2 to 9 per treatment group) were: 20.5 ± 0.5 (Vehicle/No *C. difficile*), 21.5 ± 0.7 (Vehicle/*C. difficile*/No Vanco), 22.4 ± 0.6 (Vehicle/*C. difficile*/Vanco) and 22.1 ± 0.5 (BC30/*C. difficile*/Vanco). There were no statistically significant differences in net body weight gains during the study (days 6 to 17).

### BC30 treatment significantly improved the stool consistency in C. difficile infected mice

**Figure 3:**
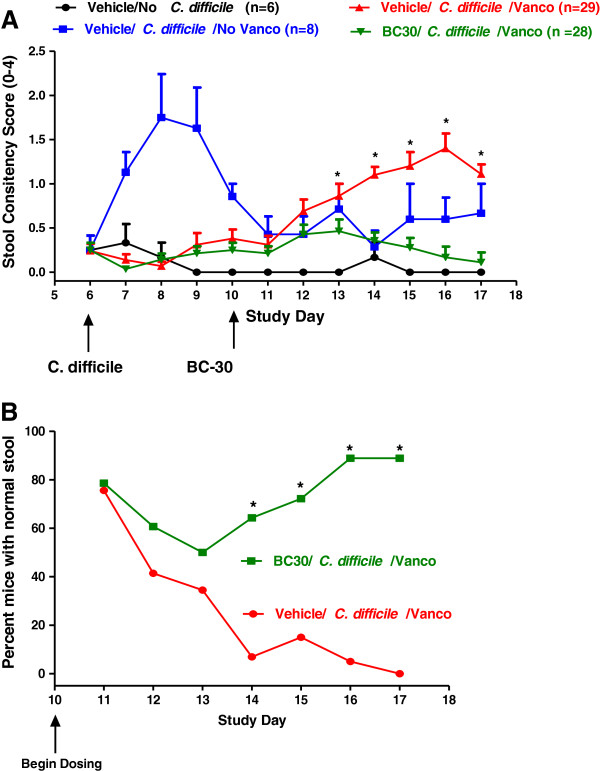
**Stool consistency data. A**) Mice were randomized on study day 6 to one of four treatment groups. All mice in the negative control group (n=6) that did not receive *C. difficile* (black symbols, lines) generally had normal stools throughout the study. Stool consistency scores were higher in the group of mice (blue symbols, bars) that were treated with Vehicle/*C. difficile*/No Vanco. In these animals, disease was prominently present on days 7 to 9. For the other two experimental groups, mice received Vehicle/*C. difficile*/Vanco and either Vehicle (red symbols, lines) to induce disease recurrence (study days 11 to 17), or BC30 at a dose of 2 x 10^9^ CFU per day (green symbols, lines). * indicates p < 0.05 vs. BC30/*C. difficile*/Vanco treatment group on study days 13 through 17. **B**) The percentages of mice with normal stools in the Vehicle/*C. difficile*/Vanco (red symbols, lines) and BC30/*C. difficile*/Vanco (green symbols/lines) treatment groups is shown in this panel. Data are shown for study days 10 through 17. On days 14 through 17, significant differences (* p < 0.05) were found in the percentages of mice with normal stools in the Vehicle/BC30/Vanco group as compared to the Vehicle/C. difficile/Vanco group. On day 17, 88.9% of mice treated with BC30 had normal stools while this value was 0% with vehicle treatment.

Figure 3 illustrates the effects of BC30 treatment on stool consistencies in *C.difficile* treated mice. The mean stool consistency score in Vehicle/*C. difficile*/Vanco treated mice (red symbols and lines) increased from 0.4 (day 10) to a range of 1.1 to 1.4 for days 14 to 17. This increase in stool consistency score indicates the recurrence of colitis. In contrast, during this time period, there was virtually no increase in the mean stool consistency score of BC30/*C. difficile*/Vanco treated mice (green symbols and lines). The stool consistency scores were significantly lower in this cohort of animals (p < 0.05 vs. Vehicle/*C. difficile*/Vanco treatment) on study days 13 through 17 [Figure 3A].

In Figure 3B, a significant difference (p<0.05) in the percentage of mice with normal stools was evident in the BC30/*C. difficile*/Vanco group, as compared to the Vehicle/*C. difficile*/Vanco group, on days 14 to 17. On day 17, 88.9% of mice treated with BC30 had normal stools compared to 0% of mice with normal stools in the Vehicle treated animals (p=0.0004 vs. Vehicle).

Stool sizes (lengths, with higher numbers indicative or more normal stools) in mm (n = 2 to 18 per group) were: 6.9 ± 0.6 (Vehicle/No *C. difficile*), 5.7 ± 0.6 (Vehicle/*C. difficile*/No Vanco), 5.9 ± 0.6 (Vehicle/*C. difficile*/ Vanco) and 7.4 ± 0.4 (BC30/*C. difficile*/Vanco). However, there were no statistically significant differences in stool sizes between treatment groups.

### BC30 treatment improved biochemical and histological indices of recurrent CDAD in mice

**Figure 4:**
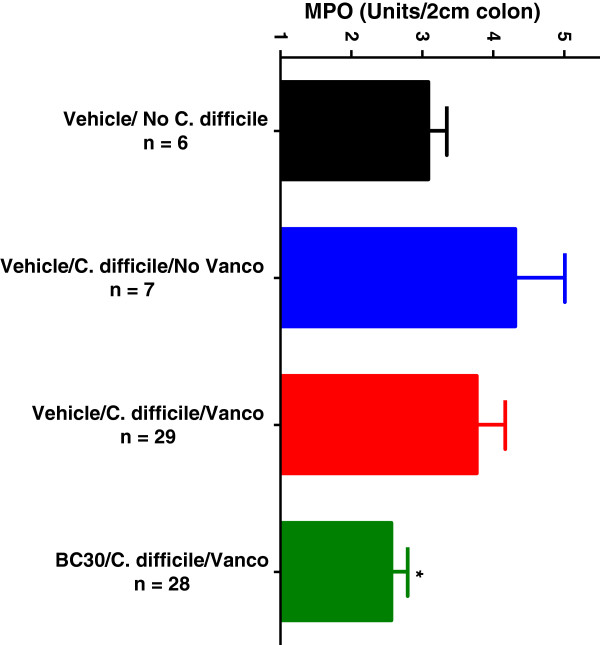
**Colonic myeloperoxidase.** Measurements of colonic myeloperoxidase (MPO) levels for all mice are shown as Units per 2 cm of colon. Colonic MPO was significantly (p < 0.05) reduced from 4.3 ± 0.7 (Vehicle/Vanco, red bar) to 2.6 ± 0.2 (BC30/Vanco, green bar).

Colonic myeloperoxidase (MPO) was measured with colonic samples from study days 14, 16, and 17. As shown in Figure 4, MPO (Units/2 cm colon) was significantly reduced (p < 0.05) from 4.3 +/−0.7 (Vehicle/*C. difficile*/Vanco treatment) to 2.6 +/−0.2 (BC30/*C. difficile*/Vanco treatment).

**Figure 5:**
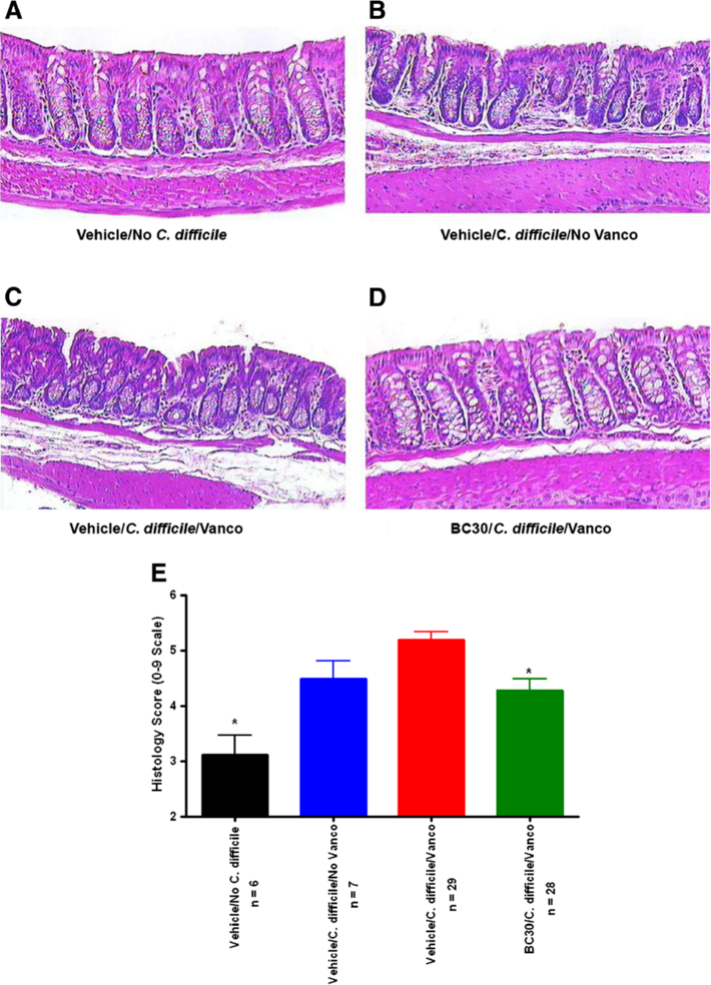
**Colonic histology.** Representative histology pictures from hematoxylin and eosin (H&E) stained colonic specimens are shown at a magnification of 200-fold. **A**) A relatively normal histological appearance is evident in the colon from a mouse not infected with *C. difficile.***B**) Evidence of crypt damage, submucosal edema and the influx of inflammatory cells in the lamina propria and sub-mucosa is present in the colon of an animal infected with C. difficile but not treated with Vanco. **C**) In the colon of a mouse given *C. difficile* plus Vanco, there is evidence of crypt disruption, leukocyte influx and prominent sub-mucosal edema. **D**) Mild pathology is observed in the colon of a BC30 treated mouse that was also given *C. difficile* plus Vanco. Modest leukocyte influx is present in the lamina propria, as well as limited sub-mucosal edema, when compared to the vehicle control (compare panels **C** and **D**). **E**) This panel shows a summary of the colonic histology score data. * p < 0.05 vs. Vehicle/*C. difficile*/Vanco treatment group (compare red and green bars in the graph).

Representative colonic histology pictures are shown in Figure 5. *C. difficile* infection, without subsequent Vanco administration, caused altered colonic histopathology. Specifically, some crypt damage as well as modest submucosal edema and moderate influx of inflammatory cells into the lamina propria and sub-mucosa were evident in the colon of this mouse (panel B). In a somewhat similar fashion, Vehicle/*C. difficile*/Vanco treated mice had clear evidence of histological pathology, including significant sub-mucosal edema (panel C). Overall, BC30 treatment (panel D) resulted in a significant improvement of the altered colonic histological pathology, which was observed in the Vehicle/*C. difficile* cohort of animals (panel C). The mean colonic histology scores were: 3.12 ± 0.35 (Vehicle/No *C. difficile*), 4.49 ± 0.32 (Vehicle/*C. difificile*/*No Vanco*), 5.19 ± 0.15 (Vehicle/*C. difificile*/*Vanco*) and 4.29 ± 0.20 (BC30/*C. difficile*/Vanco). Of note, there was a significant reduction (p< 0.05) in the mean histology score of BC30/*C. difficile*/Vanco treated mice, as compared to Vehicle/*C. difificile*/Vanco treated animals (Figure 5E).

The KC (keratinocyte derived chemokine) results (pg/2 cm colon) for all cohorts of mice were: 18.6 ± 1.2 (Vehicle/No *C. difficile*), 26.1 ± 4.3 (Vehicle/*C. difficile*/*No Vanco*), 20.8 ± 2.8 (Vehicle/*C. difificile*/*Vanco*) and 18.6 ± 1.9 (BC30/*C. difficile*/Vanco). Generally, colonic KC levels were higher in both *C. difficile*/No Vanco and *C. difficile*/Vanco treated mice. In contrast, the BC30/*C. difficile*/Vanco treatment group had a colonic KC content that was equivalent to mice that were not infected with *C. difficile*. However, there were no statistically significant differences between any of the treatment groups.

**Figure 6:**
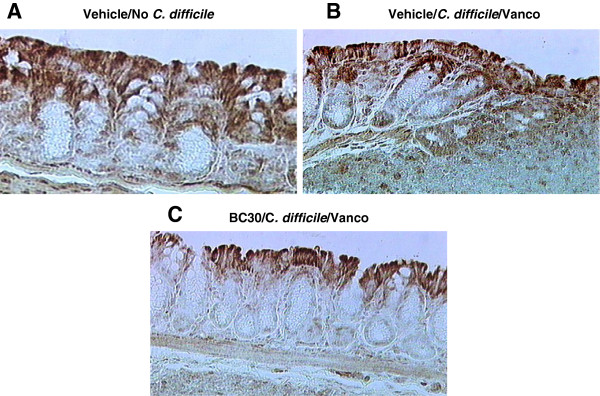
**Colonic COX-2 immunohistochemistry.** COX-2 immunohistochemistry was performed on representative histology slides from colonic samples of three treatment groups (panels **A**, **B** and **C**). As shown in **panel A,** Immuno-staining for COX-2 was evident primarily in the colonic epithelial cells from a mouse that was not infected with *C. difficile*. In the colon of a Vehicle/*C.difficile*/Vanco treated animal there was prominent brown COX-2 staining in colonocytes, as well as infiltrating leukocytes within the lamina propria and submucosa (panel **B**). Only minimal COX-2 immuno-staining (i.e., primarily in surface colonic epithelial cells) was present within the colon of a BC30/*C. difficile*/Vanco treated mouse (panel **C**).

Some representative colonic COX-2 immunohistochemistry pictures are shown in Figure 6. Interestingly, immuno-staining for COX-2 was evident primarily in the colonic epithelial cells from a mouse that was not infected with *C. difficile* (panel A). In the colon of a Vehicle/*C. difficile*/Vanco treated animal there was prominent brown COX-2 staining in colonocytes, as well as infiltrating leukocytes within the lamina propria and submucosa (panel B). Of note, only minimal COX-2 immuno-staining (i.e., primarily in surface colonic epithelial cells) was present within the colon of a BC30/*C. difficile*/Vanco treated mouse (panel C).

## Discussion

Other investigators have described the recurrence of CDAD following Vanco withdrawal in mice [10,19]. Chen et al. reported severe recurrent CDAD in mice following the removal of Vanco. CDAD was associated with severe diarrhea, prominent body weight loss, marked histological pathology, and a 58% mortality rate [10]. In contrast, Sun and colleagues found only mild diarrhea, transient body weight loss, and no evidence of mortality following Vanco withdrawal in mice. It should be mentioned that different strains of C. difficile (VPI10463 or UK 101) were used in the two studies, as well as somewhat different Vanco treatment regimens [10,11]. Despite the fact that we used the same strain of *C. difficile* (VPI10463) as Chen and colleagues, our mortality and stool consistency results (Figure 3) are more similar to those reported by Sun et al. [11]. Differences in these study results may also be related to alterations in endogenous bacterial flora populations within the colonies of mice. Certain types of bacteria that predominate in the colon (e.g., numbers of Firmicutes and Proteobacteria) have recently been shown by other investigators to critically influence the severity of *C. difficile* induced colitis in mice [20].

Interestingly, our results suggested that treatment of mice with BC30 slightly lowered the overall C*. difficile* infection rate (Figure 2A), as well as the measured levels of associated toxins in the stool (Figure 2B). However, statistically significant differences were not found compared to the corresponding cohort of vehicle treated animals. These results suggest the possibility that BC30 probiotic treatment may have lowered the actual numbers of *C. difficile* in the colonic lumen and/or mucosa. However, more detailed follow-up studies would be needed to critically test this possibility.

Previously, we found that pre-treatment of mice with B30 improved the stool consistency during the primary phase of *C. difficile* infection [18]. In a similar fashion, our results show that BC30 treatment significantly improved both the stool consistency scores and percentage of mice with normal stools (Figure 3) during the recurrence phase (days 11–17) following Vanco withdrawal in mice. Of note, mice treated with BC30 tended to have longer and firmer stools (increased stool size) than Vehicle/*C. difficile* treated mice. These results re-affirm the positive effects of this probiotic on stool consistency (Figure 3).

Other laboratories have found that toxin A secreted by *C. difficile* can activate the NF-κB and AP-1 signal transduction system in monocytes and colonic epithelial cells [6,8,21]. This process leads to secretion of a key pro-inflammatory chemokine (IL-8) and subsequent neutrophil influx into the colonic tissue [6,8,21]. Interestingly, BC-30 can significantly inhibit IL-8 directed migration of human neutrophils *in vitro*[17]. Based on these results, we measured the effects of BC30 on colonic MPO, as well the murine chemokine (KC) content in the colons of *C. difficile* infected mice. Probiotic treatment resulted in a significant reduction in colonic MPO (Figure 4), as well as a diminution in the KC content. However, statistical significance was not achieved for reducing this chemokine, as compared to values in vehicle treated mice. Nevertheless, these positive effects of BC30 on parameters associated with neutrophil influx into the colon may also contribute to the observed improvement in stool consistency observed in the probiotic-treated mice.

Murine CDAD is associated with a specific colonic histopathology that includes crypt damage, submucosal edema and influx of inflammatory cells [10]. These pathological changes were also evident during the recurrence phase in our Vehicle/*C. difficile*/Vanco treated mice (panel C, Figure 5). Interestingly, histological pathology also persisted to some degree in the Vehicle/C. difficile/ No Vanco cohort of mice (panel B, Figure 5), even at 8 to 11 days after the initial infection with *C. difficile*. In contrast, mice treated with BC30 showed evidence of improved colonic histopathology, including decreased leukocyte influx into the colon and diminished sub-mucosal edema (panel D, Figure 5). Importantly, the comparisons of mean colonic histology scores showed a statistically significant reduction in B30 treated mice compared to the corresponding vehicle cohort of animals (Figure 5E).

Other investigators have found evidence of *in vitro* and *in vivo* COX-2 induction in colonocytes or macrophages following exposure to *C. difficile* derived toxin A [9,22]. Moreover, inducible COX-2 may contribute through prostaglandin formation to the alteration in stool consistency that is a prominent feature of CDAD [10,18]. Therefore, it is interesting that colonic COX-2 immunostaining was dramatically diminished in the colon of BC30 treated mice (Figure 6). It is possible that this probiotic may affect the CREB-COX-2-PGE2 pathway, which promotes fluid secretion and contributes to CDAD in mice [9,10,18]. Future studies could focus on more critically evaluating the effects of BC30, as well as other *Bacillus coagulans* probiotic strains, on this important pathway of CDAD.

## Conclusions

BC30 limited the recurrence of *CD*-induced colitis following vancomycin withdrawal in mice. Specifically, this probiotic significantly improved stool consistency of mice in this recurrence model of CDAD. BC30 also significantly attenuated histological and biochemical indices (MPO) of infectious colitis.

## Methods

### *Bacillus coagulans* GBI-30, 6086 (GanedenBC^30^)

BC30 and maltodextrin were obtained from Ganeden Biotech Inc. (Mayfield Heights, OH).

### *Clostridium difficile* (VPI 10463)

VPI 10463 was obtained from Dr. Efi Kokkotu, (Beth Israel Deaconess Medical Center, Boston, MA) and ATCC (Manassas, VA).

### Mice

Male C57 Bl/6 mice (≈ 9 weeks of age) were purchased from Jackson Laboratory (Bar Harbor, ME). Mice were acclimated in our research facility for approximately 3 to 4 weeks, before use in experimental studies.

### Murine *Clostridium difficile*-Induced colitis

The protocol for *Clostridium difficile* recurrence developed by Chen et al. was followed with slight modifications [10]. Briefly, an antibiotic cocktail (kanamycin (0.4 mg/mL), gentamicin (0.035 mg/mL),colistin (850 U/ml), metronidazole (0.215 mg/mL), and vancomycin (0.045 mg/mL).was given in the drinking water to mice on study days 0 to 3. Subsequently, clindamycin (10 mg/kg) was administered to mice by a single i.p. injection. On study day 6, mice were randomized to receive VPI 10463 (≈ 5 x 10^4^ CFU) by oro-gastric gavage. A negative disease control group of animals was administered vehicle (0.9% saline). Subsequently, on day 6, mice received either vancomycin (50 mg/kg) or 0.9% saline (vehicle) by oro-gastric gavage, until day 10. On study day 10, animals were randomized to receive either BC30 (2 x 10^9^ CFU per day), or vehicle (50% maltodextrin in 0.9% saline), which were dosed by oro-gastric gavage until study day 16. Both body weight and stool consistency data were collected daily on study days 10 through 17. Stool samples from all mice were scored based on the consistency of the fecal sample, as shown here: 0 = normal, 1 = loose stool, 2 = loose/some diarrhea, 3 = diarrhea and 4 = severe watery diarrhea [18].

Based on preliminary time course studies, mice were euthanized on days 14, 16, or 17 (i.e., cohorts 1, 2 or 3) in Figure 1. On these study days, we confirmed the presence of *Clostridium difficile* and associated toxins (A and B) in stools with a Wampole^TM^ CD quick check complete kit from TECHLAB (Blacksburg, VA). Furthermore, the amount of toxins A and B in available stool samples was determined in a semi-quantitative fashion by use of a *C. DIFFICILE TOX A/B II*^TM^ ELISA KIT from TECHLAB (Blacksburg, VA). Also, in some mice, stool size (length in mm) was determined with electronic callipers from available specimens.

On these same study days (days 14, 16 or 17), mice were euthanized; and the distal colon was collected for evaluating morphometric (colon weight), histological and biochemical parameters. An overview of the study design is shown in Figure 1. This study was repeated twice and results were combined in the final data analyses. Since no significant differences in measured parameters of CDAD were found on study days 14, 16, and 17, these data were combined for data analyses. The protocol was approved by the Institutional Animal Care and Use Committee (IACUC) at the Penn State College of Medicine.

### Colonic histology evaluation

Using coded slides from the distal colon, four areas from each slide were scored on a three-point severity scale: 0 = Normal, 1 = Mild, 2 = Moderate, 3 = Severe, for three different parameters. These three parameters were epithelial damage, mucosal/submucosal edema and leukocyte infiltration. Therefore, the total score for each slide (i.e., mouse) was between 0 and 9 [18]. Histology photographs (H&E staining) were captured at 200x magnification using an Olympus IMT-2 microscope (Olympus Corporation, Lake Success, NY) and EPIX-XCAP® image capture software (Buffalo Grove, IL).

### Colonic MPO

Colonic myeloperoxidase (MPO) was utilized as an indicator of neutrophil influx into the mouse colon, as described previously by our laboratory [18]. Results were expressed as Units/2 cm colon.

### Colonic KC (CXCL1) chemokine content

KC (keratinocyte derived chemokine) is a functionally relevant murine chemokine [7]. The colonic KC content was measured with an ELISA kit from R&D systems (Minneapolis, MN). Results are expressed as pg/2 cm colon.

### COX-2 Immunohistochemistry: Mouse colon

Generally, we followed the procedures for immunohistochemistry with colonic tissue samples, which have been described previously by our laboratory [23]. For the cyclooxygenase-2 (COX-2) primary antibody, we used a 200-fold dilution, as suggested by the manufacturer (Cell Signaling, Danvers, MA). Representative, COX-2 immunohistochemistry photographs from mouse colons were captured at a 300x magnification, using the aforementioned Olympus IMT-2 microscope and EPIX-XCAP® image capture software program.

### Statistical analyses

All statistical analyses were performed with a GraphPad Prism® (San Diego, CA). Differences in the percentages of mice with normal stools, as well as percentages of mice with *C. difficile* infection were determined with the Fisher’s exact test. Stool consistency scores were evaluated by the Mann Whitney test. Biochemical and histological data were evaluated using unpaired t test analyses. A p value of < 0.05 was considered to be statistically significant for all parameters.

### Ethical statement

This study, which utilized mice, was approved by the IACUC at the Penn State College of Medicine. The corresponding author was involved in the intellectual aspects of the study. GanedenBC^30^ is a patented strain of Ganeden Biotech Inc. All requests to use GanedenBC^30^ for further research should be made directly to the company and are evaluated on an individual basis.

## Abbreviations

BC30: *Bacillus coagulans* GBI-30, 6086; KC: Keratinocyte derived chemokine; CDAD: *Clostridium difficile*-associated disease; CDI: Clostridium *difficile* infection.

## Competing interests

None of the authors have any conflict of interest disclosures to make regarding this manuscript, with the exception of Sean Farmer and Dr. David Keller. Sean Farmer and Dr. Keller are paid employee of Ganeden Biotech Inc.

## Authors’ contributions

LRF contributed to the technical and intellectual aspects of the manuscript. WHG, KDK, SF and DK contributed to the intellectual aspects of the paper. JSS contributed to the technical aspect of the manuscript. All the authors read and approved the manuscript.
